# Thoracic Motion Analysis Using a TrueDepth Camera in Patients with Relapsing Polychondritis: A Pilot Study

**DOI:** 10.3390/healthcare13212664

**Published:** 2025-10-22

**Authors:** Yoshihiro Nishi, Shohei Sato, Hiroshi Handa, Hiroki Nishine, Takemi Matsui, Masamichi Mineshita

**Affiliations:** 1 Division of Respiratory Medicine, Department of Internal Medicine, St. Marianna University School of Medicine, Kawasaki 216-8511, Japan; hiroshihstv@marianna-u.ac.jp (H.H.);; 2 Department of Electrical Engineering and Computer Science, Graduate School of Systems Design, Tokyo Metropolitan University, Tokyo 191-0065, Japan; sato.shohei@tmu.ac.jp (S.S.);

**Keywords:** non-invasive respiratory assessment, relapsing polychondritis, smartphone-based health monitoring, spirometry, thoracic motion analysis

## Abstract

**Background/Objectives**: Relapsing polychondritis (RP) is a rare autoimmune disorder marked by recurrent inflammation of cartilaginous tissues, including the airways. Airway involvement, such as subglottic stenosis and airway malacia, significantly impacts prognosis. Although spirometry is the standard for evaluating respiratory function, it may be unfeasible in patients with severe airway narrowing or tracheostomy. This study evaluated the potential of a smartphone-based application, DepthRecorder, which uses the iPhone’s TrueDepth camera to analyze thoracic motion in real time. **Methods**: Twelve patients with RP were enrolled. All underwent simultaneous respiratory assessment using spirometry and the DepthRecorder application. Thoracic motion data were corrected for height using previously validated regression formulas. Correlation between DepthRecorder and spirometry values was analyzed using Spearman’s rank correlation for forced vital capacity (FVC), forced expiratory volume in one second (FEV_1_), and the FEV_1_/FVC ratio. **Results**: Mean age was 53.8 ± 13.3 years, with equal numbers of males and females. Before correction, DepthRecorder showed moderate correlations for FEV_1_ (ρ = 0.48, *p* = 0.003) and FEV_1_/FVC (%) (ρ = 0.57, *p* < 0.001). After correction, stronger correlations were observed for FVC (ρ = 0.76, *p* < 0.001), FEV_1_ (ρ = 0.72, *p* < 0.001), and FEV_1_/FVC (%) (ρ = 0.60, *p* < 0.001). **Conclusions**: The DepthRecorder application demonstrated strong correlations with spirometry following height-based correction. This method may offer a practical, non-invasive tool for respiratory assessment in RP patients who cannot undergo conventional lung function testing. Further studies are needed to validate these findings and establish clinical reference standards.

## 1. Introduction

Relapsing polychondritis (RP) is a rare, autoimmune disorder characterized by recurrent inflammation and destruction of cartilaginous tissues. Airway involvement, such as subglottic stenosis, central airway stenosis, and airway malacia, significantly impacts disease progression and patient outcomes [1]. Standard treatment options typically include systemic corticosteroids, immunosuppressive agents, and biologics [2]. In severe cases of subglottic stenosis, tracheostomy may be required. For patients with airway malacia, non-invasive or invasive positive pressure ventilation can provide temporary relief; however, interventional bronchoscopy may become necessary when airway obstruction is unresponsive to these methods [3].

Spirometry remains the gold standard for evaluating airway obstruction, but it may be impractical or impossible in patients with tracheostomy or severe airway compromise. Imaging tools such as computed tomography (CT) and positron emission tomography (PET)/CT have shown utility in assessing airway structure but pose limitations due to radiation exposure and cost [4]. A previous report showed chest CT parameters may serve as a spirometry alternative, though frequent assessment remains difficult.

Several studies have investigated thoracic motion as a non-invasive method of assessing respiratory function. Kaneko et al. measured thoracic cage and abdominal wall movements in heathy subjects using a 3D motion analysis system, reporting the influence of age, posture, and gender on movement amplitude [5]. Similarly, Blanco-Almazán et al. modeled the contribution of thoracic movement and respiratory volume to thoracic bioimpedance, showing that a combined model yielded minimal error [6]. These studies support the potential of thoracic motion analysis as a tool for respiratory assessment.

Recently, a non-contact early airflow limitation screening system (EAFL-SS) was developed that enables home-based respiratory evaluation [7]. This prototype used a time-of-flight (ToF) depth sensor to visualize anterior thoracic motion. Previously, a noninvasive, noncontact method using depth sensor was reported in patients with central airway stenosis [8]. Based on these prior findings, the present study investigated the feasibility of using the TrueDepth camera, which employs a structured light system, to assess anterior thoracic motion in patients with RP. The objective was to evaluate whether the DepthRecorder application could serve as a non-invasive alternative to spirometry in cases where conventional lung function testing is not feasible.

## 2. Materials and Methods

### 2.1. Patient and Public Involvement

Patients or the public were not involved in the design, conduct, reporting or dissemination plans of this research.

### 2.2. Study Design and Participants

This prospective study enrolled 12 patients with RP who attended the outpatient clinic at St. Marianna University Hospital between October and December 2024. All participants underwent simultaneous respiratory assessments using both conventional spirometry and the DepthRecorder application, which analyses thoracic motion.

### 2.3. Device and Measurements

Measurements were obtained using a smartphone-based application, DepthRecorder, which captures anterior thoracic movements in real time via the TrueDepth camera on an iPhone 13 Pro^®^. The application was based on principles adapted from the previously developed EAFL-SS system, although the TrueDepth camera uses a structured light system rather than a ToF sensor.

**Figure 1:**
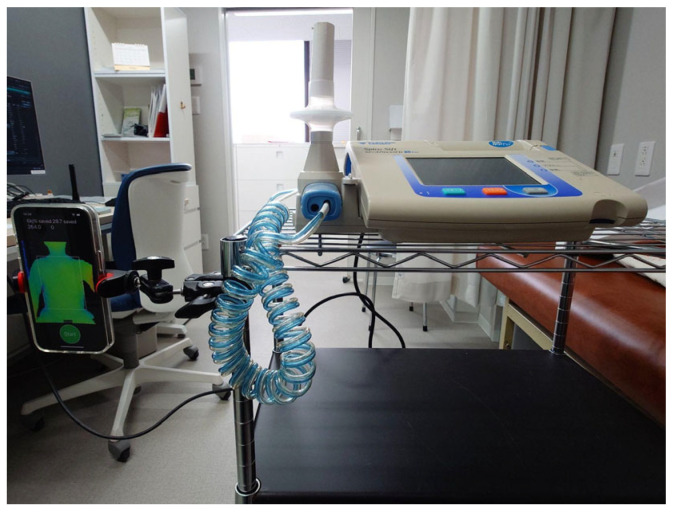
Equipment setup showing simultaneous spirometry and DepthRecorder application display. The photo illustrates the measurement environment used in the study, including the spirometer and an iPhone 13 Pro^®^ displaying the DepthRecorder application interface. The subject sits approximately 30–40 cm from the iPhone while both spirometry and thoracic motion data are recorded simultaneously.

**Figure 2:**
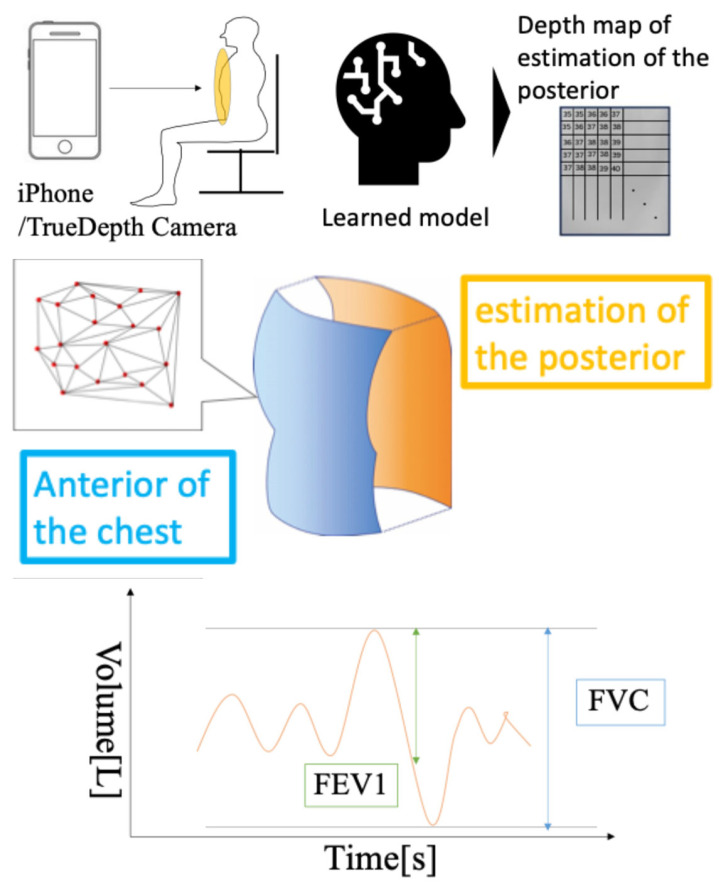
Schematic workflow of the study. The diagrams illustrate measurement of anterior thoracic motion using the iPhone TrueDepth camera, subsequent analysis of the captured data, and graphical processing to estimate forced vital capacity (FVC) and forced expiratory volume in one second (FEV_1_).

The examinee was instructed to sit 35 cm from the device using the LCD screen as a guide. An iPhone 13 Pro^®^ with the DepthRecorder installed is shown in Figure 1. When the TrueDepth camera detected the correct sitting position, DepthRecorder automatically displayed an image of the examinee’s anterior thorax on the screen. Measurements were initiated by pressing the “Start Measurement” button on the app, simultaneously with the start of spirometry. A schematic overview of the measurement process is shown in Figure 2.

**Figure 3:**
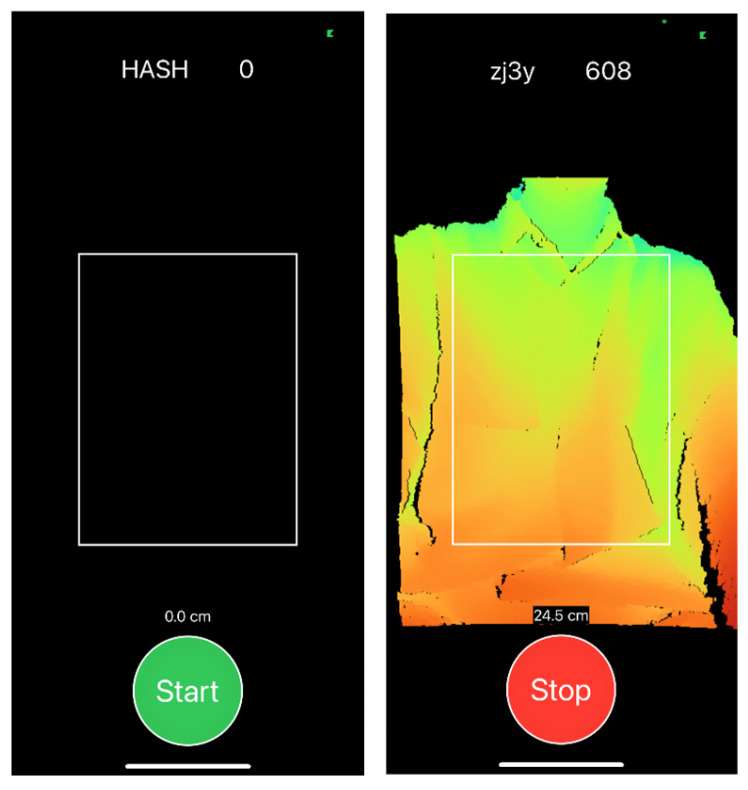
Screenshots of the DepthRecorder application before and during measurement. The left screenshot shows the DepthRecorder interface on the iPhone screen before the measurement begins. The right screenshot captures the ongoing measurement process, where the participant’s torso is visualized in real time using the TrueDepth camera.

A thoracic bounding box detection algorithm was used to extract the thorax region from the captured image [9]. The anterior thoracic depth image was binarized to remove background and objects located more than 15 cm from the initial reference plane. The algorithm also removed non-thoracic areas such as the neck and head. Readings were considered invalid if any object or individual other than the participant entered the visual field of the iPhone (Figure 3). Sampling and analysis were performed using LabView 2019 Professional Development System software (National Instruments, Austin, TX, USA).

Respiratory function was simultaneously assessed using a standard spirometer (FUKUDA SP-370 Hyper, Tokyo, Japan), and measurements with DepthRecorder were taken during the same breath cycle. All measurements were performed in a seated position on a backless chair in an examination room, with three sets of readings per patient. Data were analyzed at Tokyo Metropolitan University.

### 2.4. Statistical Analysis

All analyses were performed to evaluate the correlation between spirometry values and respiratory measurements obtained using the DepthRecorder application. Spearman’s rank correlation coefficient was used to assess associations for three parameters: forced vital capacity (FVC), forced expiratory volume in one second (FEV_1_), and the FEV_1_/FVC ratio (%).

To improve accuracy and comparability, we applied a correction formula derived from multiple regression analysis in prior research involving 48 patients with COPD and 19 healthy individuals [10]. Corrected DepthRecorder FVC (mL) was calculated as −3050.49 + 28.69 × height (cm) + 1.667 × measured FVC (mL). Corrected DepthRecorder FEV_1_ (mL) was calculated as −2109.28 + 17.82 × height (cm) + 2.180 × measured FEV_1_ (mL). These corrected DepthRecorder values were then compared with corresponding spirometry data using Spearman’s rank correlation. Due to the small sample size and the presence of both mild and severe cases, we analyzed data using the nonparametric Spearman’s correlation. Spearman’s rank correlation coefficient is a widely used non-parametric measure for assessing the monotonic relationship between two variables [11].

### 2.5. Ethical Considerations

This study was approved by the Biomedical Research Ethics Committee of St. Marianna University School of Medicine (approval No.: 5557, approval date: 21 February 2022). All participants provided written informed consent prior to participation.

## 3. Results

Twelve patients with RP were included in the analysis. Two of the 12 patients had a history of asthma, and none had undergone chest surgery. The mean age was 53.8 ± 13.3 years, with six male and six female participants. The average height was 162.3 ± 10.3 cm, and the average weight was 58.3 ± 12.1 kg. Patient characteristics and respiratory function measurements are summarized in Table 1.

Abbreviations: FVC, forced vital capacity; FEV_1_: forced expiratory volume in one second; FEV_1_/FVC (%), forced expiratory volume in one second divided by forced vital capacity, expressed as a percentage.

**Figure 4:**
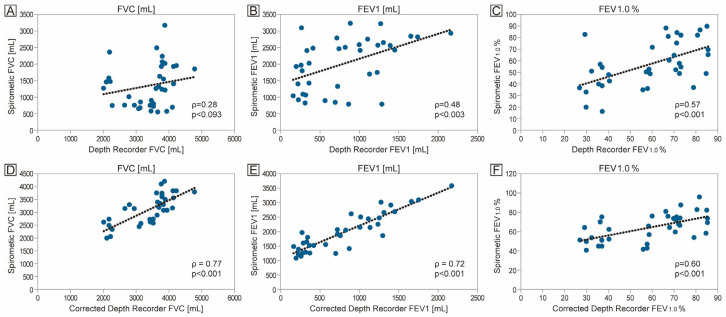
Correlation between DepthRecorder and spirometry values before and after correction. Scatter plots illustrate the relationship between DepthRecorder-derived values and spirometric readings in patients with relapsing polychondritis. Panels (**A**–**C**) show the uncorrected correlations for forced vital capacity (FVC), forced expiratory volume in one second (FEV_1_), and the FEV_1_/FVC ratio (%), respectively. Panels (**D**–**F**) show the corresponding correlations after applying a height-based correction formula for FVC, FEV_1_, and FEV_1_/FVC ratio (%).

Figure 4A–C show the correlation between uncorrected DepthRecorder values and standard spirometry. Before correction, moderate correlations were observed for FEV_1_ (ρ = 0.48, *p* = 0.003) and FEV_1_/FVC (%) (ρ = 0.57, *p* < 0.001), while the correlation for FVC was not statistically significant.

After correction for height using validated regression formulas, stronger correlations were observed with spirometry, as shown in Figure 4D–F. Corrected DepthRecorder FVC (mL) and corrected DepthRecorder FEV_1_ (mL) showed stronger correlations with spirometry measurements. The corrected DepthRecorder FVC (mL) and corrected DepthRecorder FEV_1_ (mL) were significantly correlated with spirometric measurements for FVC (ρ = 0.76, *p* < 0.001), FEV_1_ (ρ = 0.72, *p* < 0.001), and FEV_1_/FVC (%) (ρ = 0.60, *p* < 0.001).

## 4. Discussion

This study observed significant correlations between spirometry values and DepthRecorder measurements, corrected for height, in 12 patients with airway involvement due to RP. These results suggest that DepthRecorder may serve as a viable alternative for respiratory function assessment, particularly in patients who are difficult to evaluate using conventional spirometry, such as those with tracheostomies or severe bronchial stenosis. These findings support the potential of this smartphone-based tool as a non-invasive alternative for respiratory assessment. Further research is required to refine the methodology, validate its performance in larger populations, and establish robust clinical reference standards.

Previous studies have utilized 3D depth-sensing technologies to estimate respiratory function through thoracic motion analysis [12,13]. While some systems, such as those used by Takamoto et al., employed ToF sensors [7], the TrueDepth camera used in this study is based on a structured light system that also uses infrared light to capture three-dimensional surface geometry. Takamoto et al. evaluated a ToF sensor in 32 COPD patients and 21 healthy controls, demonstrating strong correlation with spirometry during forced expiration while seated. The reported sensitivity and specificity for detecting early airflow limitation were 81% and 90%, respectively, with FEV_1_ values closely correlated with spirometry. These findings reinforce the utility of 3D scanning technologies, both ToF and structured light systems, for non-contact respiratory assessment. Depth cameras such as Microsoft Kinect and Intel RealSense use dot projector-based measurement methods, which are fundamentally the same as those employed by the iPhone TrueDepth camera. In preliminary experiments, we used iPhones to measure cylindrical artificial objects and found the accuracy of volume calculations to be sufficiently high. The main sources of error in this study are more likely related to the non-linear relationship between thoracic volume and lung capacity or to inter-individual variability. Accordingly, differences in equipment are unlikely to have exerted a dominant influence on the results.

The use of the TrueDepth camera offers the additional benefit of smartphone integration, potentially enabling remote and non-invasive monitoring of respiratory function. Such applications may be especially beneficial during home-based care or online consultations, where traditional lung function tests are impractical. For patients with RP or severe COPD who face mobility challenges, the ability to assess respiratory status from home may facilitate earlier detection of exacerbations and timely intervention. Moreover, this method may aid intraoperative assessment of airway status.

A previous report demonstrated the utility of thoracic motion analysis before and after treatment in supine-positioned patients with airway stenosis, indicating potential intraoperative applications when endoscopic procedures are required [8]. Given the heterogeneity of airway involvement in RP, conventional respiratory tests might not fully reflect disease severity, highlighting the need for alternative approaches such as the one presented here. Smartphones enable users to perform measurements without specialized equipment, allowing simple respiratory function testing even outside clinical facilities. This may facilitate easier monitoring of disease progression and support telemedicine applications. However, a drawback is that body movement can easily affect measured volumes, leading to unstable readings and underscoring the need for further improvements in the measurement method.

There were several limitations in this study. First, measurement variability remains challenging, influenced by factors including body movement during forced exhalation, the inability to capture posterior thoracic motion in the seated position, and interference from loose clothing. Additionally, thoracic contour and body habitus may affect measurement accuracy. Finally, the small sample size limits the generalizability of the findings. Because the correction formulas were externally derived and uniformly applied, new multivariable regression was not performed, which limited our ability to assess the contribution of individual predictors. The regression equations were based only on the starting position and the FVC and FEV_1_ values one second later. Therefore, the accuracy of the conversion at other points on the flow–volume curve cannot be guaranteed, and the overall shape of the curve may differ from that obtained with spirometry.

## 5. Conclusions

The measurements obtained using the DepthRecorder were corrected using equations derived from multiple regression analysis based on preliminary experimental results. The corrected DepthRecorder values demonstrated significant correlations with spirometry: FVC (ρ = 0.76, *p* < 0.001), FEV_1_ (ρ = 0.72, *p* < 0.001), and FEV_1_/FVC (%) (ρ = 0.60, *p* < 0.001). These results indicate strong agreement with spirometry in patients with airway involvement due to RP. The findings support the potential of this smartphone-based tool as a non-invasive alternative for respiratory assessment, particularly when conventional spirometry is not feasible. Further research is required to refine the methodology, validate its performance in larger and more diverse populations, and establish robust clinical reference standards.

## Figures and Tables

**Table 1:** Participant characteristics and respiratory function measurements by spirometry and DepthRecorder before and after correction.

Age	53.8 ± 13.3
Gender (m/f)	6/6
Height (cm)	162.3 ± 10.3
Weight (kg)	58.3 ± 12.1
Lesion	
Tracheobronchial	6
Trachea	1
Main bronchi	3
None	2
Spirometry	
FVC (mL)	3630 ± 696.77
FEV_1_ (mL)	2220 ± 797.31
FEV_1_/FVC (%)	63.9 ± 19.41
TrueDepth camera	
FVC (mL)	1303 ± 650.81
FEV_1_ (mL)	739 ± 521.92
FEV_1_/FVC (%)	53.85 ± 19.77
Corrected DepthRecorder	
FVC (mL)	3152 ± 581.01
FEV_1_ (mL)	1906 ± 658.44
FEV_1_/FVC (%)	65.05 ± 14.11

Data are presented as mean ± standard deviation (SD).

## Data Availability

The raw data supporting the conclusions of this article will be made available by the authors on request.

## Abbreviations

The following abbreviations are used in this manuscript:

RP	Relapsing polychondritis
CT	Computed tomography
PET	Positron emission tomography
EAFL-SS	Early airflow limitation screening system
ToF	Time-of-flight
COPD	Chronic obstructive pulmonary disease
FVC	Forced vital capacity
FEV_1_	Forced expiratory volume in one second
FEV_1_/FVC (%)	Ratio of forced expiratory volume in one second to forced vital capacity

## References

[1] Trentham D.E., Le C.H. (1998). Relapsing polychondritis. Ann. Intern. Med..

[2] Petitdemange A., Sztejkowski C., Damian L., Martin T., Mouthon L., Amoura Z., Cutolo M., Burmester G.R., Fonseca J.E., Rednic S. (2022). Treatment of relapsing polychondritis: A systematic review. Clin. Exp. Rheumatol..

[3] Yin R., Xu D., Wang Q., Li M., Zhang W., Zhang F., Zeng X., Jiang N., Hou Y. (2024). Predictors and prognosis of tracheostomy in relapsing polychondritis. Rheumatology.

[4] Tsuruoka H., Handa H., Yamashiro T., Nishine H., Inoue T., Mineshita M. (2021). Correlation between computed tomographic analysis and pulmonary function measurements in patients with relapsing polychondritis. Respiration.

[5] Kaneko H., Horie J. (2012). Breathing Movements of the Chest and Abdominal Wall in Healthy Subjects. Respir Care.

[6] Blanco-Almazán D., Groenendaal W., Catthoor F., Jané R. (2019). Chest Movement and Respiratory Volume both Contribute to Thoracic Bioimpedance during Loaded Breathing. Sci Rep..

[7] Takamoto H., Nishine H., Sato S., Sun G., Watanabe S., Seokjin K., Asai M., Mineshita M., Matsui T. (2020). Development and clinical application of a novel non-contact early airflow limitation screening system using an infrared time-of-flight depth image sensor. Front. Physiol..

[8] Mitsuya M., Nishine H., Handa H., Mineshita M., Kurosawa M., Kirimoto T., Sato S., Matsui T., Sun G. (2025). Spatiotemporal chest wall movement analysis using depth sensor imaging for detecting respiratory asynchrony. Inform. Med. Unlocked.

[9] Ostadabbas S., Sebkhi N., Zhang M., Rahim S., Anderson L., Lee F., Ghovanloo M. (2016). A vision-based respiration monitoring system for passive airway resistance estimation. IEEE Trans. Biomed. Eng..

[10] Tanaka S. Assessment of pulmonary function using TrueDepth camera in patients with COPD. Proceedings of the European Respiratory Society Congress.

[11] Zar J.H. (2005). Spearman rank correlation. Encyclopedia of Biostatistics.

[12] Soleimani V., Mirmehdi M., Damen D., Dodd J., Hannuna S., Sharp C. (2017). Remote, depth-based lung function assessment. IEEE Trans. Biomed. Eng..

[13] Taeger J., Bischoff S., Hagen R., Rak K. (2021). Utilization of smartphone depth mapping cameras for app-based grading of facial movement disorders: Development and feasibility study. JMIR Mhealth Uhealth.

